# A short-term increase of the postoperative naturally circulating dendritic cells subsets in flurbiprofen-treated patients with esophageal carcinoma undergoing thoracic surgery

**DOI:** 10.18632/oncotarget.7669

**Published:** 2016-02-24

**Authors:** Di Wang, Xin-lu Yang, Xiao-qing Chai, Shu-hua Shu, Xiao-lin Zhang, Yan-hu Xie, Xin Wei, Yu-jing Wu, Wei Wei

**Affiliations:** ^1^ Department of Anesthesiology, Affiliated Provincial Hospital of Anhui Medical University, Anhui Provincial Hospital, Hefei 230001, China; ^2^ Institute of Clinical Pharmacology, Anhui Medical University, Hefei 230032, China

**Keywords:** dendritic cells, flurbiprofen, PGE2, esophageal carcinoma, esophageal resection surgery

## Abstract

The present study evaluated whether flurbiprofen increased the naturally circulating dendritic cells (DCs) subsets in patients with esophageal squamous cell carcinoma (ESCC) undergoing esophageal resection. Compared to healthy donors (n=20), the significantly depressed percentages of plasmacytoid DCs (pDCs), CD1c^+^ myeloid DCs (mDCs), and CD141^+^ mDCs among ESCC patients (n=60) were confirmed. Flurbiprofen was administered before skin incision and at the end of operation in group F (n=30), as well as placebo in group C (n=30). The postoperative suppressed percentages of pDCs, CD1c^+^ mDCs, and CD141^+^ mDCs increased significantly following the perioperative treatment with flurbiprofen. Flurbiprofen also significantly stimulated the postoperative IFN-f and IL-17 production, but inhibited the immunosuppressive IL-10 and TGF-β levels. Furthermore, flurbiprofen exerted a similar analgesic effect and brought a significantly less sufentanil consumption compared to group C. Taken together, flurbiprofen provided a short-term increase of postoperative naturally circulating DCs in ESCC patients.

## INTRODUCTION

Despite rare occurrence among peripheral blood mononuclear cells (PBMCs), naturally circulating dendritic cells (DCs) display a strikingly strong ability in taking up, processing, and presenting pathogens or tumor-associated antigens (TAAs) to stimulate naive T cells and to induce cytotoxic T lymphocytes (CTLs) or helper 1 T cells (Th1)/Th17 polarization [1, 2]. Recently, a novel DCs-based vaccination strategy, which employed enriched naturally circulating DCs directly isolated from PBMCs, showed a impressive clinical efficacy in patients with prostate cancer and melanoma, strongly suggesting a potent ability of naturally circulating DCs to induce the specific anti-cancer CTLs and Th1/Th17 response [3, 4]. The perioperative treatment with cyclooxygenase (COX) inhibitors, such as non-steroidal anti-inflammatory drugs (NSAIDs), not only alleviate postoperative pain and reduce opioid analgesics consumption, but also enhance anti-tumor immunity, particularly in T cell subsets and associated cytokines-dependent anti-tumor immunity [5, 6]. In the present study, we investigated whether flurbiprofen increased postoperative naturally circulating DCs subsets in patients with esophageal squamous cell carcinoma (ESCC) undergoing esophageal radical resection.

## RESULTS

### Naturally circulating DCs subsets in healthy donors and ESCC patients

**Figure 1:**
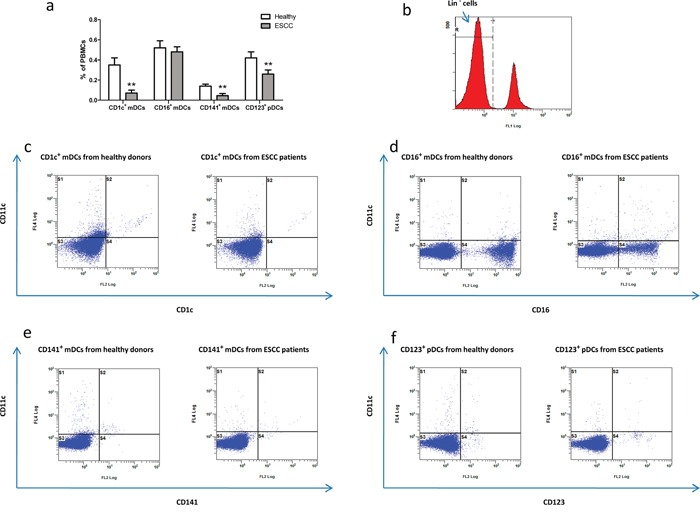
The percentages of circulating DCs subsets among PBMCs in healthy donors and ESCC patients The percentages of circulating DCs subtypes depicted in the histogram in **a.** Lin^−^ PBMCs as a gating strategy in **b.** Specifically, the representative FACS plots of CD11c^+^ CD1c^+^ in **c.** CD11c^+^ CD16^+^ in **d.** CD11c^+^ CD141^+^ in **e.** CD11c^−^ CD123^+^ in **f.** 10,000 events were acquired for each sample. ** *P*<0.01 versus healthy controls.

Traditionally, circulating DCs among PBMCs was detected by characterization of negative for lineage (Lin) markers (CD3, CD14, CD19, CD20, CD56) and positive for HLA-DR. The Lin^−^ HLA-DR^+^ cells can be further subdivided into mDCs and pDCs by the expression of CD11c and CD123, respectively. Recently, some novel and potent circulating DCs subtypes, including CD1c^+^ mDCs (Lin^−^ CD11c^+^ CD1c^+^), CD16^+^ mDCs (Lin^−^ CD11c^+^ CD16^+^), and CD141^+^ mDCs (Lin^−^ CD11c^+^ CD141^+^), among naturally circulating mDCs (Lin^−^ HLA-DR^+^ CD11c^+^) were reported [1, 2]. The percentages of CD1c^+^ mDCs, CD16^+^ mDCs, CD141^+^ mDCs and CD123^+^ pDCs among PBMCs in healthy donors and ESCC patients were preoperatively (pre-OP) evaluated by fluorescence activated cell sorting (FACS) and depicted as the histogram (Figure 1a). Specifically, CD1c^+^ mDCs (Figure 1c), CD141^+^ mDCs (Figure 1e) and CD123^+^ pDCs (Figure 1f) were significantly suppressed compared to the healthy controls. But except for CD16^+^ mDCs, CD16^+^ mDCs percentage in ESCC patients was similar with healthy control (Figure 1d).

### Flurbiprofen increased postoperative circulating DCs subsets in patients

**Figure 2:**
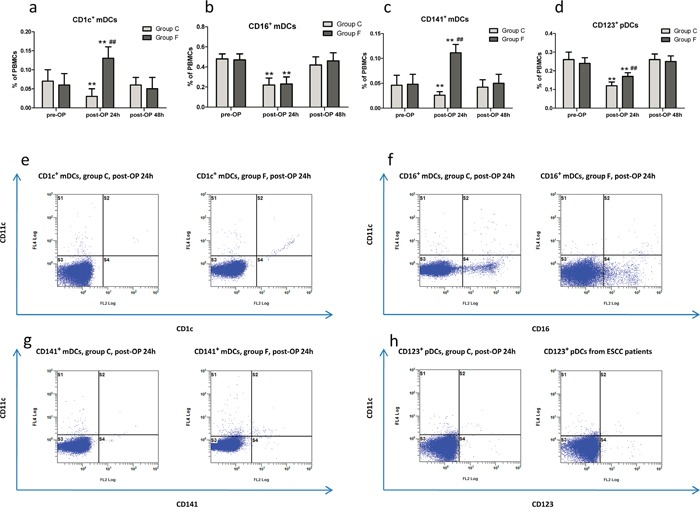
The percentages of pDCs and three mDCs subtypes among PBMCs at pre-OP, post-OP 24h and 48h from patients in group F and C, respectively **a-d.** Representative FACS plots of DCs subtypes at post-OP 24h **e-h.** **P<0.01 versus pre-OP baseline levels. ^##^*P*<0.01 versus patients received placebo in group C.

The percentages of pDCs and mDCs subtypes among PBMCs from patients were determined by FACS at postoperatively (post-OP) 24h and 48h, respectively. In group C, the percentages of pDCs and three mDCs subtypes at post-OP 24h were significantly depressed compared to pre-OP baseline levels, and no significant difference in the four DCs subsets percentage between post-OP 48h and pre-OP baseline levels. Comparing to group C, the percentages of CD1c^+^ mDCs (Figure 2e), CD141^+^ mDCs (Figure 2g) and CD123^+^ pDCs (Figure 2h) at post-OP 24h were up-regulated significantly in group F after perioperative treatment with flurbiprofen, except for CD16^+^ mDCs (Figure 2f). At following post-OP 48h, the four DCs subsets percentage between group F and group C did not show a significant change. In group F, the percentages of CD1c^+^ mDCs (Figure 2a) and CD141^+^ mDCs (Figure 2c) at post-OP 24h were significantly increased compared to pre-OP baseline levels, and the percentages of CD16^+^ mDCs (Figure 2b) and CD123^+^ pDCs (Figure 2d) at post-OP 24h were significantly suppressed compared to pre-OP baseline levels, and the four DCs subsets percentage returned to pre-OP baseline levels at post-OP 48h.

### Flurbiprofen promoted the postoperative anti-tumor cytokine profile

**Figure 3:**
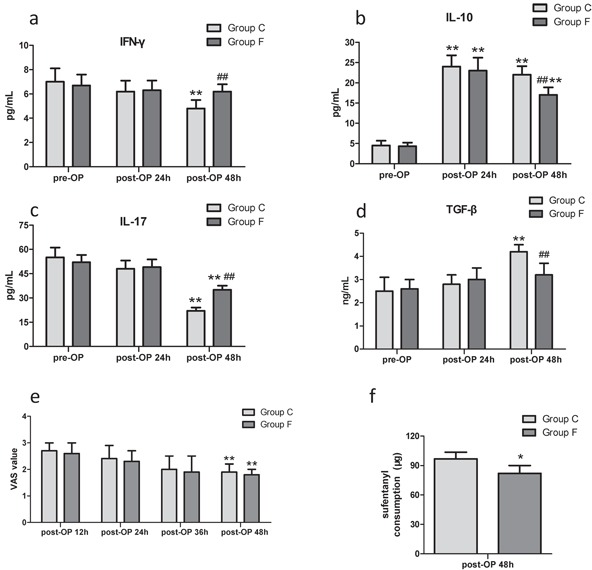
IFN-γ and IL-17 levels in group F and group C **in a and c.** IL-10 and TGF-β levels in group F and group C in **b** and **d.** **P<0.01 versus pre-OP baseline level. ^##^*P*<0.01 versus patients received placebo in group C. No significant difference in VAS scores between groups in **e.** **P<0.01 versus post-OP 12h. Sufentanil consumption during post-OP 24h in group F was significantly less in **f.** *P<0.05 versus patients received placebo in group C.

In group C, the cancer-inhibitory cytokine IFN-γ and Th17-associated IL-17 production were significantly suppressed at post-OP 48h compared to pre-OP baseline levels (Figure 3a, 3c). Yet the immunosuppressive cytokines IL-10 and TGF-β levels at post-OP 48h were significantly higher than pre-OP baseline levels in group C (Figure 3b, 3d). Notably, flurbiprofen significantly increased anti-cancer IFN-γ and IL-17 levels at post-OP 48h in group F (Figure 3a, 3c). Accordingly, IL-10 and TGF-β levels at post-OP 48h were depressed significantly in patients from group F following perioperative treatment with flurbiprofen (Figure 3b, 3d).

### Flurbiprofen brought a similar analgesia and less sufentanil consumption

During post-OP 48h patient-controlled intravenous analgesia (PCIA), there was no significant difference in visual analogue scale (VAS) scores between group C and group F (Figure 3e). The opioid analgesics sufentanil consumption during post-OP 24h in group F was significantly less than group C (Figure 3f). The incidence of nausea/vomiting between group C and group F did not show a significant change (data not shown).

## DISCUSSION

Previously, not only numerical defective of circulating DCs and tumor-infiltrating DCs in tissue specimen, but also functional impairment of monocyte-derived DCs (moDCs) from the peripheral blood was reported in patients with esophageal and other tumors [7–10]. In the present study, FACS results showed that CD1c^+^ mDCs represented by far the most predominant mDCs subset (0.35±0.07%, Figure 1c) in the healthy donors (except for CD16^+^ mDCs), whereas CD141^+^ mDCs (0.14±0.02%, Figure 1e) formed a minute population. Specifically, CD1c^+^ mDCs and CD141^+^ mDCs can be activated by a distinct set of toll-like receptors (TLRs), and subsequently secrete large amounts of cytokines IFN-γ and IL-12 which allow the highly effective induction of CTLs and Th1 responses against tumor [11, 12]. In line with the defective quantification of DCs in esophageal carcinoma, FACS results in the present study displayed that the percentages of CD1c^+^ mDCs, and CD141^+^ mDCs among PBMCs in ESCC patients were significantly lower than healthy controls. However, the similar percentages of CD16^+^ mDCs subset, considered to be more monocyte-like, among PBMCs were identified in healthy donors and ESCC patients. Besides mDCs, pDCs are specialized in the detection and control of viral infections, as well as prime both Th1 and CTLs by the strong release of IFN-γ in an IL-12-independent Th1 polarization [13, 14]. Moreover, several studies demonstrated that despite lower antigen uptake and limited phagocytosis than mDCs subsets, pDCs isolated from blood and spleen were shown to efficiently cross-present antigens and prime potent tumor-specific CTLs [15–17]. Thus, pDCs, as well as CD1c^+^ mDCs, represent a promising target in DCs-based vaccine immunotherapy for cancer. But unfortunately, similar to CD1c^+^ mDCs, the FACS results showed that pDCs percentage (0.26±0.04%, Figure 1f) among PBMCs from ESCC patients was also significantly depressed compared to healthy control (0.42±0.06%, Figure 1f).

The COX-derived prostaglandin E2 (PGE2), a well-known tumor-sustaining inflammatory mediator, greatly contributes to tumorigenesis, angiogenesis, metastasis in malignances through successful evasion of tumor immune surveillance and resistance to cancer immunotherapy [18–20]. A growing amount of information supports that tumor-derived PGE2 specifically exerts a suppressive effect on DCs biology through a paracrine and/or autocrine manner, which reduces the maturation of DCs and their expression of MHC class II molecules, ability to present TAAs and prime anti-tumor T cells, possibly via enhancing the production of immunosuppressive IL-10 [21–23]. Although data not shown in the present study, the previous researches demonstrated that the surgery operations significantly induced the over-production of PGE2 perioperatively, which greatly contributed to further disruption of the impaired immune function of cancer patients [24, 25]. In the present study, FACS and ELISA results showed that the postoperative naturally circulating DCs subtypes and anti-tumor cytokines profile were markedly decreased mainly due to the surgical trauma and pain-induced the immune suppression via immunosuppressive mediators release, such as PGE2, IL-10, TGF-β. Subsequently, these naturally circulating DCs subtypes returned to pre-OP baseline levels shortly after post-OP 48h, possibly related to opioid analgesics usage for alleviating the postoperative pain and/or limited life expectancy of circulating DCs. Given the immunosuppressive role of PGE2 in cancer progression, a series of researches have provided evidence on the clinical beneficial of NSAIDs which inhibit increased COX activity and PGE2 production on tumor progression, particularly in a remarkable synergy between NSAIDs and CTLA-4 or PD-1 blockade immunotherapy results in tumor eradication [20, 26, 27]. Accordingly, research found that the preoperative treatment with NSAIDs (indomethacin or celecoxib) was enough to increase tumor infiltration and peripheral blood by seemingly activated immune cells in patients with colorectal carcinoma and gastric cancer [6, 20, 28]. The tumor-specific COX ablation stimulated tumor-infiltrating CD11c^+^ MHC-II^+^ DCs displayed higher levels of co-stimulatory molecules, and over-production of PGE2 impaired accumulation of DCs within melanoma and suppress their activation, including IL-12-producing activity [20]. In the present study, our results focused on perioperative treatment with flurbiprofen significantly up-regulated the postoperative percentages of CD1c^+^ mDCs, CD141^+^ mDCs and CD123^+^ pDCs, as well as anti-tumor cytokine profile, but this short-term increase of the postoperative naturally circulating DCs subsets in flurbiprofen-treated patients didn't last for post-OP 48h. This might be explained by the short elimination half-life time of flurbiprofen in plasma (approximately 5.8 h) [29, 30].

In the present study, we preliminarily observed the perioperative prevalence of naturally circulating DCs among PBMCs, and assessed the impact of flurbiprofen on the postoperative naturally circulating DCs percentages in the ESCC patients. Although, we found that the postoperative 3-month survive rate of patients between group F and group C did not show a significant change after esophageal resection (data not shown), whether the short-term increase of the circulating DCs subsets in flurbiprofen-treated patients effectively provides an improved long-term prognosis with ESCC patients still needs consistent follow-up. What's more, we are currently investigating the effect of PGE2/flurbiprofen on the function of enriched naturally circulating DCs *ex vivo*, such as phagocytic activity, co-stimulatory molecule expression, antigen presenting ability and cytokines production. In summary, the present study provided a better understanding of the anti-tumor role of naturally circulating DCs and promotion of PGE2 on tumor immune evasion in esophageal cancer, and suggested that NSAIDs could be useful additions to potential circulating DCs-based immune therapy or conventional treatment of cancer patients.

## MATERIALS AND METHODS

### Patients

The protocol of this study was approved by the Ethics Committee of Anhui Medical University and the Chinese Clinical Trial Registry (No.ChiCTR-IPR-15006482). Written informed consent was obtained from all subjects. 60 patients with ESCC undergoing esophageal resection with an American Society of Anesthesiologists (ASA) status of I-II were enrolled, as well as 20 age-matched healthy donors. The enrolled patients, whom met the inclusion criteria in the present study, ranged in age from 50 to 75 years and weighed from 45 to 80kg (summarized in Table 1). The exclusion criteria were as follows: (1) allergy to NSAIDs, (2) blood coagulation disorder, (3) hepatic or renal dysfunction, (4) autoimmune disease or acute inflammation, (5) perioperative blood transfusion, (6) bronchial asthma, (7) preoperative treatment with radiotherapy, chemotherapy, immunodepressant, or glucocorticoid.

**Table 1:** Characteristics of patients enrolled in the present study

	Patients in group F (n=30)	Patients in group C (n=30)	Healthy donors (n=20)
**Age (year)**	56.45±5.29	57.12±5.58	56.82±5.37
**Gender (male/female)**	27/3	28/2	17/3
**Weight(kg)**	64.95±8.26	63.35±8.72	62.97±6.95
**ASA (I/II)**	27/3	26/4	
**Surgery time (min)**	303.26±29.59	298.65±28.65	
**Infusion fluid (ml)**	1945.00±250.21	1935.00±279.61	
**Urine output (ml)**	493.00±75.26	485.00±63.04	
**Blood loss (ml)**	281.50±63.27	297.00±49.96	

### Group and treatments

The ESCC patients were randomly assigned to either the flurbiprofen treatment group (group F, n=30) or the control group (group C, n=30). For patients in group F, flurbiprofen axetil (1mg/kg, i.v., 50mg/5ml, Beijing Tide Pharmaceutical, China) was administered 15 min before skin incision and at the end of surgery, respectively. The placebo (intralipid, Chengdu Huarui Pharmaceutical, China) was administered at the same time points in patients from group C. Both two groups were equally received postoperative PCIA using sufentanil (100μg).

### Anesthesia and analgesia

General anesthesia was induced with 0.05mg/kg midazolam, 2mg/kg propofol, 0.4μg/kg sufentanil, and 1.0mg/kg rocuronium. A left double-lumen endobronchial tube (Mallinckrodt, Ireland) was inserted and confirmed by fiberoptic bronchoscopy. Mechanical ventilation was performed using an anesthesia machine (S/5 Avance, Datex-Ohmeda, USA) with tidal volume (V_T_) ranged in 6-8 ml/kg and positive end-expiratory pressure (PEEP) 5cmH_2_O, and was adjusted to maintain the P_ET_CO_2_ at 35-45mmHg. The inspiratory to expiratory time (I/E) ratio was 1:2. During the surgery, 50μg/kg/min propofol and 0.1-0.2μg/kg/min remifentanil were target-controlled infused to maintain anesthesia, and cis-atracurium was simultaneously given to muscle relaxation.

### Sample collection and cytokines measurement

Patients blood sample were collected from the central venous catheters before anesthesia, as well as post-OP 24h and 48h. Plasma from venous blood was obtained by centrifugation (1000×*g*, 4°C, 5 min) and stored at −80°C for further analyses. Cytokines IFN-γ, IL-10, IL-17, TGF-β levels in plasma were determined using the ELISA kits (R&D, CA, USA) according to the manufacturer's instructions. PBMCs were separated from peripheral blood by Ficoll-Hypaque density gradient centrifugation and re-suspended in 0.2 ml PBS (4×10^6^/100μl) for further FACS study.

### Circulating DCs subsets measured by FACS

As shown in Figure 1b detection of naturally circulating DCs subsets was achieved by CD1c^+^ mDCs (CD11c^+^ CD1c^+^), CD16^+^ mDCs (CD11c^+^ CD16^+^), CD141^+^ mDCs (CD11c^+^ CD141^+^), and CD123^+^ pDCs (CD11c^−^ CD123^+^) within a Lin^−^ PBMCs gate. Following mouse monoclonal antibodies (mAbs) were used in the present study, including FITC anti-human Lin cocktail (Biolegend, US), the anti-human CD11c-APC, anti-human CD1c-PE, anti-human CD16-PE, anti-human CD141-PE, anti-human CD123-PE, and respective mouse IgG isotype controls (Miltenyi, Germany). The absolute counts of pDCs and three mDC subsets were quantitated as the percentages of them among PBMCs measuring by Cytomics FC500 Dual Laser System (Beckman Coulter, US).

### PCIA clinical assessment

PCIA efficacy was evaluated at post-OP 12h, 24h, 36h, and 48h according to the VAS scores, including: 0 (painless), 1-4 (mild pain), 5-8 (moderate pain), and 9-10 (severe pain). Sufentanil consumption and incidence of nausea/vomiting during post-OP 48h were recorded.

### Statistical analysis

Statistics were analyzed with the SPSS software (version 16.0). Quantitative data are presented as means ± SD. Significant differences were evaluated by Student's t-test or ANOVA. *P* values less than 0.05 were considered statistically significant.
